# Neuroimaging data repositories and AI-driven healthcare—Global aspirations vs. ethical considerations in machine learning models of neurological disease

**DOI:** 10.3389/frai.2023.1286266

**Published:** 2024-02-19

**Authors:** Christine Lock, Nicole Si Min Tan, Ian James Long, Nicole C. Keong

**Affiliations:** ^1^Department of Neurosurgery, National Neuroscience Institute, Singapore, Singapore; ^2^Duke-NUS Medical School, Singapore, Singapore

**Keywords:** data repositories, neuroimaging, machine learning, artificial intelligence (AI), AI ethics

## Abstract

Neuroimaging data repositories are data-rich resources comprising brain imaging with clinical and biomarker data. The potential for such repositories to transform healthcare is tremendous, especially in their capacity to support machine learning (ML) and artificial intelligence (AI) tools. Current discussions about the generalizability of such tools in healthcare provoke concerns of risk of bias—ML models underperform in women and ethnic and racial minorities. The use of ML may exacerbate existing healthcare disparities or cause post-deployment harms. Do neuroimaging data repositories and their capacity to support ML/AI-driven clinical discoveries, have both the potential to accelerate innovative medicine and harden the gaps of social inequities in neuroscience-related healthcare? In this paper, we examined the ethical concerns of ML-driven modeling of global community neuroscience needs arising from the use of data amassed within neuroimaging data repositories. We explored this in two parts; firstly, in a theoretical experiment, we argued for a South East Asian-based repository to redress global imbalances. Within this context, we then considered the ethical framework toward the inclusion vs. exclusion of the migrant worker population, a group subject to healthcare inequities. Secondly, we created a model simulating the impact of global variations in the presentation of anosmia risks in COVID-19 toward altering brain structural findings; we then performed a mini AI ethics experiment. In this experiment, we interrogated an actual pilot dataset (*n* = 17; 8 non-anosmic (47%) vs. 9 anosmic (53%) using an ML clustering model. To create the COVID-19 simulation model, we bootstrapped to resample and amplify the dataset. This resulted in three hypothetical datasets: (i) matched (*n* = 68; 47% anosmic), (ii) predominant non-anosmic (*n* = 66; 73% disproportionate), and (iii) predominant anosmic (*n* = 66; 76% disproportionate). We found that the differing proportions of the same cohorts represented in each hypothetical dataset altered not only the relative importance of key features distinguishing between them but even the presence or absence of such features. The main objective of our mini experiment was to understand if ML/AI methodologies could be utilized toward modelling disproportionate datasets, in a manner we term “AI ethics.” Further work is required to expand the approach proposed here into a reproducible strategy.

## 1 Introduction

Research collaborations within the global neurosurgical community are often hampered by differing socio-political and legal frameworks regulating the accumulation, utility, and sharing of clinical datasets. Yet, vast quantities of data are required to construct robust and meaningful clinical models for interrogation of scientific hypotheses using innovative technology-based solutions, such as Artificial Intelligence (AI)-driven tools and Machine Learning (ML) methods. ML/AI-led modeling in neurological datasets are subject to common considerations with these emerging techniques, such as lack of transparency, generalizability and risk of bias (Chen et al., 2021). In addition to geographical variations in phenotypes of neurological diseases, there are increasing challenges for the provision of neuroscience healthcare services attributed to patients with advanced age, frailty, and/or concurrent comorbidities. Such concerns provide a rich substrate for the generation of research questions to advance clinical care, but it is difficult to navigate such heterogeneity to deliver the ideals and aspirations of precision medicine. In patient populations acutely presenting for neurological interventions, baseline (i.e., previously acquired) imaging of the brain is often absent. The structural metrics of the brain are unknowable without neuroimaging. This provokes a significant gap in our understanding about how best to model patterns of reversible vs. irreversible brain injury. This capability requires that we develop neuroimaging-based data resources. Yet, as brain images may be used to simulate structural pathologies that impact upon cognitive and behavioral processes, new concerns have arisen about how these “digital twins” will be utilized within the context of global healthcare (Keong, 2021). Should the control and access to such simulated digital extensions be governed by ethical principles, such as equity and social justice, and how should vulnerable individuals and groups be represented within these initiatives?

### 1.1 Imbalances arising from neuroimaging data repositories

One response to the conundrum of lack of datasets for use in neuroscience-based healthcare is the use of neuroimaging data repositories. However, such open-access databases of de-identified brain images, often linked to anonymized data variables of clinical measures, must be disproportionately funded by high-income countries (HICs) in the pursuit of the ideals of Open Science. This reflects the maturity of such systems to (i) support legal and regulatory frameworks protecting the rights and expectations of individuals to data privacy [e.g., Health Insurance Portability and Accountability Act (HIPAA),^1^ General Data Protection Regulation (GDPR)^2^], whilst (ii) promoting solidarity in accepting personal risks to contribute to societal needs for scientific discovery. Accordingly, examples of brain repositories and data sharing initiatives [ADNI (Petersen et al., 2010), HBCD (Bakhireva et al., 2020; Morris et al., 2020), HBP (Amunts et al., 2019), CBRS (Illes et al., 2019), CONP (Poline et al., 2023), UK Biobank (Sudlow et al., 2015), etc.] are mainly from North America, Europe, and the United Kingdom (UK). A consequence of this is that HICs may be expected to reap far larger rewards from the technological advancements associated with these initiatives (such as capacity to host them, cloud computing tools for processing/analysis and tech industry-related collaborations). Gross imbalances, such as in socioeconomic factors, patient demographics and other social determinants of health, are therefore commonly found in open-access neuroimaging datasets. For example, ABIDE for autism spectrum disorder (ASD) and PHENOM for schizophrenia have 13% and 37.4% female participants, respectively (Di Martino et al., 2014; Chand et al., 2020; Wang et al., 2023). Patient groups such as those with comorbidity risk burden, as well as Asian and South-East Asian ethnicities, have been poorly represented in the cohorts of large-scale neuroimaging programs. The iSTAGING consortium dataset for Alzheimer's disease is comprised of 70.6% European Americans, 8.8% African Americans and 1.5% Asian Americans (Habes et al., 2021; Wang et al., 2023).

### 1.2 Balance between access, representation and bias when harnessing global neuroimaging data repositories for machine learning- and AI-driven discovery

#### 1.2.1 Harms

Underrepresentation of specific cohorts is a well-known problem within clinical studies and trials. It is therefore unsurprising that the same concerns regarding representation would arise in neuroimaging data repositories. The concurrent explosion of ML/AI technologies using such data-rich resources have also produced other concerns. Most repositories are funded via study or disease-specific intentions and may not have been designed to be held up as perfect mirrors of their whole communities. Even repositories built with community-based large scale needs in mind are recruited from voluntary participants. Wider societal differences in participating in initiatives may contribute to imbalances in repositories. These imbalances may reflect both inequities in access to healthcare innovations as well as reluctance by minority communities to bear societal costs of participation, e.g., potential inconveniences of time, cost, risk to personal data, or mistrust regarding the lack of benefit sharing. It is well-known that more needs to be done to ensure repositories demonstrate more proportionate representation of the spectrum of diseases and populations served by such initiatives. These efforts should be made more transparent.

The rapid rise of ML/AI methodologies leveraging on repositories have compounded concerns already present regarding such imbalances. Yet, the potential for ML/AI technologies to accelerate healthcare knowledge is undeniable. Imaging data—large, unwieldy, and not as easily processed as other forms of clinical data—are highly suited to advancements made in this field. However, the incorporation of such technologies also has the potential to increase risks of medical or clinical bias if current ML/AI methodologies are trained on incomplete datasets. These risks are 2-fold, both for the (i) consequences of incorporating ML/AI methodologies in the clinical translation of knowledge gained from neuroimaging data repositories and (ii) potential new knowledge harvest and innovations arising from the usage of these resources toward training new ML/AI technologies. Whilst it is possible, via the use of advanced ML/AI methodologies, to mitigate bias by careful training and correcting for age, sex, and ethnicity (Wang et al., 2023), nevertheless, it is still impossible to correct for the exclusion of representative cohorts or rare manifestations of disease risks (Chen et al., 2021). Known shortcomings in ML/AI techniques relate to implications in data imbalances in sex and racial disparities. There is also arguably a blind spot in healthcare, where studies have demonstrated significant variations in clinical outcomes of interventions experienced by patient cohorts of different ethnicities (Creanga et al., 2014). Concerns also arise from the implications of future utility of ML/AI methodologies being implemented in healthcare protocols with such inbuilt flaws and biases that may have potential to influence medical decision-making and outcomes (Chen et al., 2021).

#### 1.2.2 Benefits

Current patient outcomes are heavily influenced by evidence produced by clinical studies, whose non-ML methodologies are equally subject to inbuilt shortcomings. These are predicated upon the heterogeneity and inherent messiness of clinical datasets, that often contain multiple, incomplete variables with differing impact as determinants of health outcomes. There are already implicit knowledge gaps in the practice of medicine that repositories may help to address, such as geographical variations in the presentation of diseases or patient cohorts. ML/AI methodologies may better handle the problem of big datasets, such as by concurrently interrogating differing cohorts and imputing trends or plugging gaps of missing data. Whilst these shortcomings can be addressed in clinical trials via stringent inclusion and exclusion criteria, such methods only promote highly specific cohorts for research studies. If a trial is successful, it is standard practice for results to be then applied in a widespread fashion to a much broader patient group, without the threshold of previously applied criteria. This outcome may be expensive for healthcare organizations and unresponsive to specific needs of patient groups. ML/AI methodologies could create digital twin “surrogates” that could be used as disease platforms to test hypotheses or model the burden of illnesses and their potential clinical trajectories. Testing pre-clinical interventions on digital twins to improve the efficacy and cost of treatments could shorten the time needed from knowledge harvest to translatable therapies and innovative medicine. Should the questions surrounding ML/AI methodologies be solely about how they are trained and harms that could arise if their training is improperly performed? Or should more be done about rebalancing data repositories to address potential pitfalls from their utilization instead? It is clear that the ideal framework for repositories would need to balance both equitable representation within them and scientific access to such datasets, whilst considering how to mitigate potential downstream effects of bias from lack of the same. How can we approach the framework of building repositories via the lens of AI ethics to properly prepare global neuroimaging data repositories for machine learning-led innovations?

## 2 Methods

**Figure 1:**
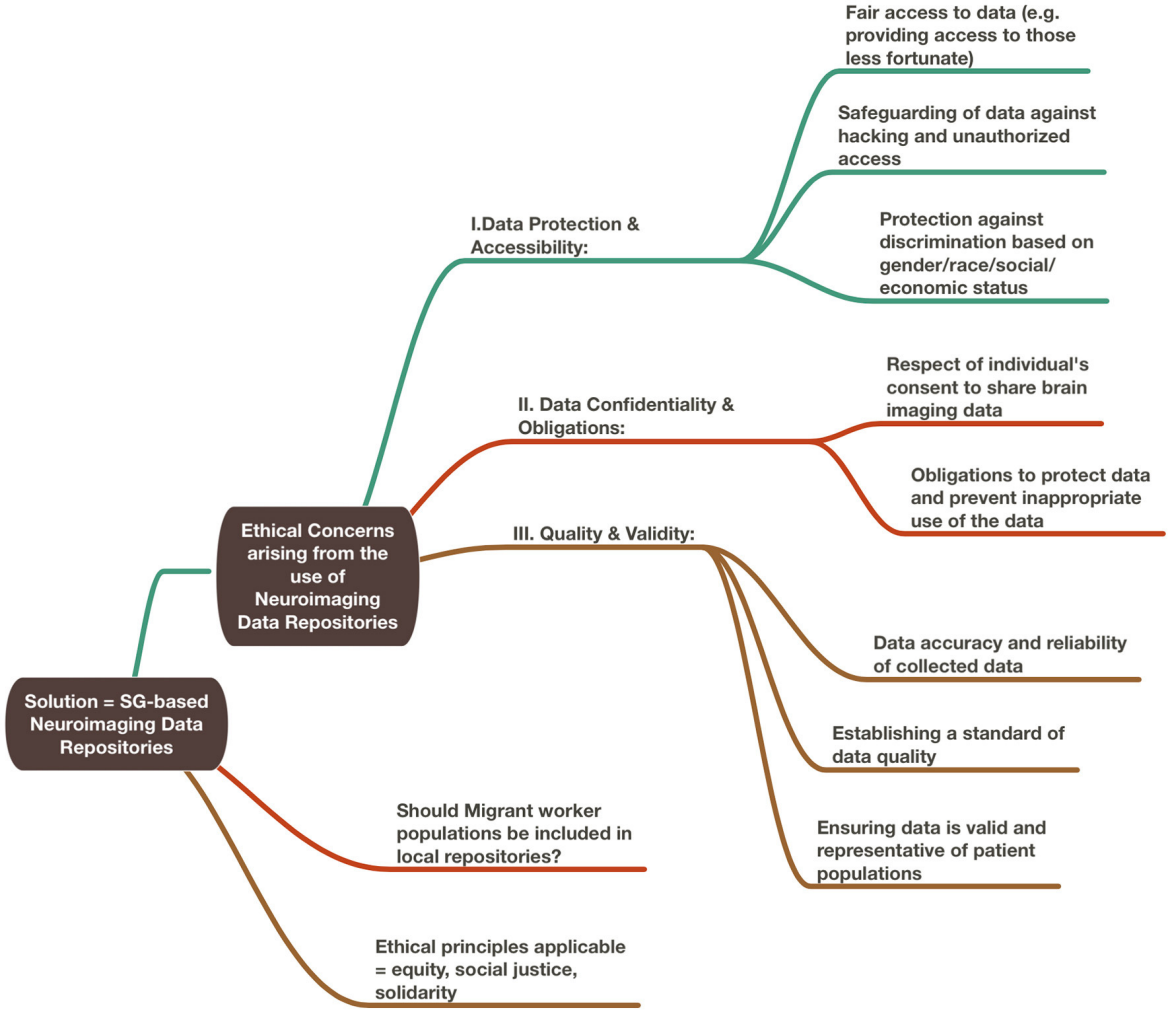
Ethical considerations arising from the use of neuroimaging data repositories.

**Figure 2:**
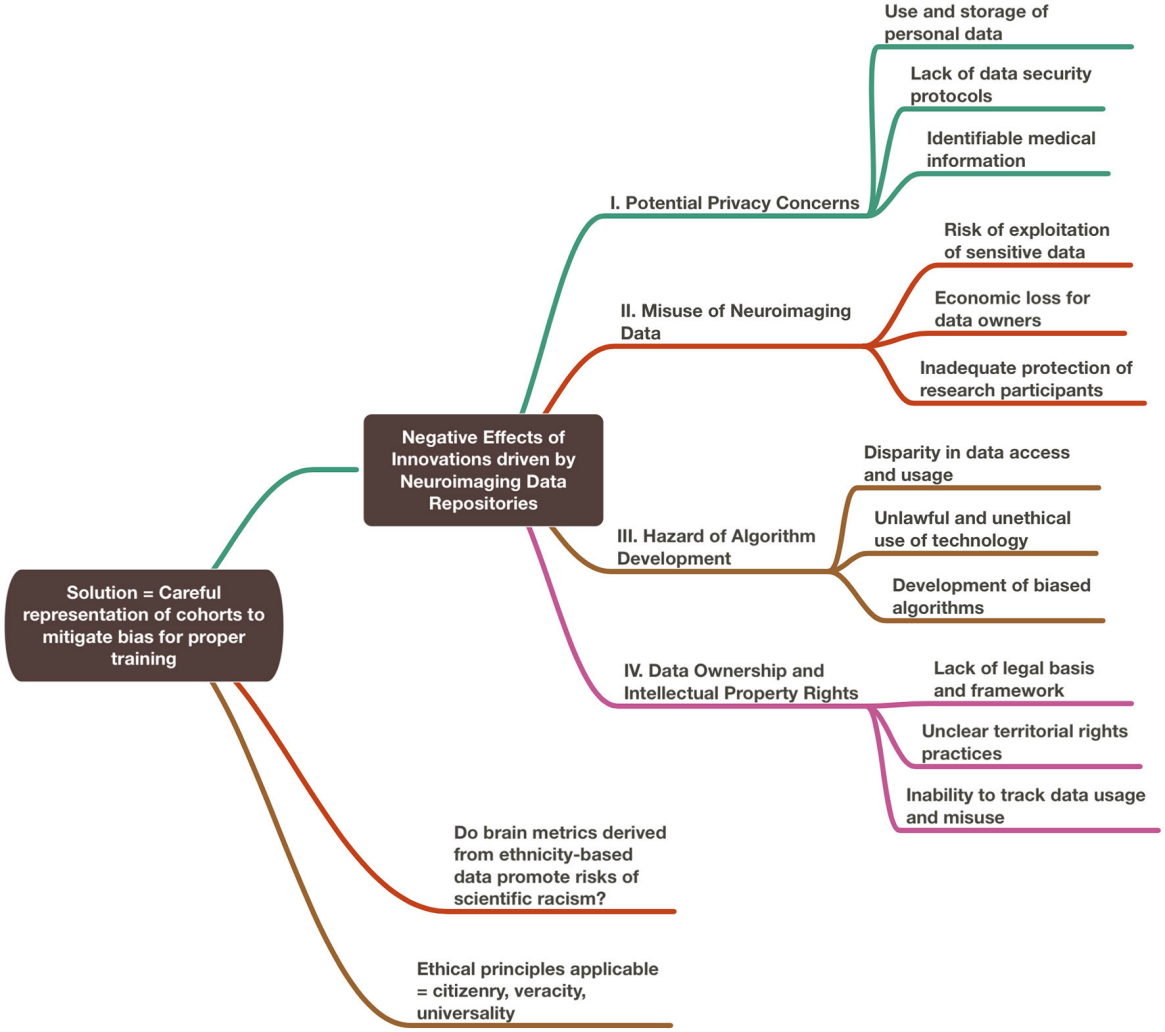
Negative effects of innovations driven by neuroimaging data repositories.

In this paper, we performed two experiments to test ethical considerations regarding the use of ML/AI-driven methodologies based on neuroimaging data repositories. In the first experiment, we considered the theoretical scenario of rebalancing repositories to mitigate risk of bias based on ethnicity-derived data. We considered whether inclusion or exclusion of the migrant worker population within a local repository best served the ethical principles that could be applied toward its governance. For this section, we incorporated the use of ChatGPT x MindNode to generate two illustrations. To do so, we performed multiple iterations of questions regarding ethical considerations surrounding the use of neuroimaging data repositories and combined the best answers generated from our ideas of topics of interest. We then edited the resulting mind maps for accuracy and clarity and added our own proposed solutions to the questions posed (Figures 1, 2).

In the second part of the paper, we performed a mini-AI ethics experiment using pilot data from an ongoing study on long COVID. Variations in COVID-19 genotype and phenotype of disease presentation have contributed to vast differences of healthcare outcomes across global populations. We examined whether or not, by perturbing the proportions of cohorts within the pilot dataset, we were able to alter findings of structural brain metrics and interpretation of brain injury patterns resulting from such analyses. We have previously published our methodologies for 3D structural segmentation and DTI profiles (Lock et al., 2019; Soon et al., 2021). For the mini AI Ethics experiment, imaging datasets were anonymized at source, then subject to further checks and deidentification, defacing and processing prior to usage, according to our lab and institutional protocols. We then performed anatomical preprocessing of the anonymized and deidentified T1-weighted (T1w) images using the fsl_anat functionality (brainlife.app.273) of the FMRIB Software Library (FSL) (Smith et al., 2004; Woolrich et al., 2009; Jenkinson et al., 2012). The anatomical T1w images were cropped and reoriented to match the MNI152 template, then subject to linear and non-linear alignment using the FLIRT tool (Jenkinson and Smith, 2001; Jenkinson et al., 2002; Greve and Fischl, 2009). We used the linearly aligned images as the acpc aligned T1w images for further post-processing using Freesurfer release 7.1.1 (brainlife.app.462) (Dale and Sereno, 1993; Sled et al., 1998; Dale et al., 1999; Fischl et al., 1999a,b, 2001, 2002, 2004a,b; Fischl and Dale, 2000; Rosas et al., 2002; Kuperberg et al., 2003; Salat et al., 2004; Ségonne et al., 2004, 2007; Desikan et al., 2006; Han et al., 2006; Jovicich et al., 2006; Reuter et al., 2010, 2012; Reuter and Fischl, 2011). The recon_all function was used to generate pial/cortical and white matter surfaces and perform brain parcellations according to known neuroanatomical atlases. We chose the Destrieux (aparc.a20090s) atlas for subsequent segmentation of white matter and mapping of diffusion metrics to the relevant cortical and subcortical structures.

Supplementary and further processing of the anonymized and deidentified images were performed using processing pipelines via the brainlife.io secure cloud processing platform, as per methodology described by Caron et al. (2021). In brief, following manual inspection of the images, additional FSL and Freesurfer steps via their equivalent brainlife apps were repeated as necessary to achieve satisfactory brain segmentation. Preprocessing of dMRI data was performed using MRTrix3, with alignment of the dMRI images to the acpc aligned T1w dataset (brainlife.app.68). MRTrix3—Anatomically-constrained probabilistic tractography (ACT) (brainlife.app.319) was used to generate white matter tractography (Smith et al., 2012; Takemura et al., 2016; Ades-Aron et al., 2018; Avesani et al., 2019; Tournier et al., 2019). Following this, cortex tissue mapping (brainlife.app.381) was performed as per Fukutomi et al. (2018) using dwi, freesurfer and tensor output from the processing steps above. Summary measures for the processing pipeline were then generated using the Freesurfer Statistics (brainlife.app.272) compute summary statistics of diffusion measures from subcortical (brainlife.app.389) and cortical segmentation (brainlife.app.483) brainlife apps for further analyses (Dale et al., 1999; Fukutomi et al., 2018; Avesani et al., 2019). At each stage of the pipeline, visual inspection was performed and manual reprocessing of individual images undertaken as needed for suboptimal output. All steps and links to open-source code for each app used are linked in Supplementary Table 1.

For the mini experiment, all brain metrics were examined qualitatively and no statistical comparisons were performed. This was due to the theoretical nature of the experiment and the hypothetical nature of the modeling. The modeling approach and ML/AI methodologies used are described in full within the section of the mini AI ethics experiment.

## 3 The theoretical experiment

In this section, we consider the provision of a technological platform that benefits low- (LIC) to middle-income (MIC) countries by proposing a thought experiment regarding the development of a South East Asian (SEA)-based neuroimaging data repository. What ethical concerns arise from this initiative to redress the balance of participants of global repositories?

### 3.1 The dilemma of underrepresented communities—Should repositories recruit participants from the local migrant worker population?

Migrant workers of varying employment categories and earning levels are governed by the sociopolitical policies of their host nations. Whilst the ethical concerns we discuss in this paper may be applicable to the broad group, workers performing low-skilled work for low wages are especially vulnerable from their participation in repository initiatives. We restrict our discussion of the issues arising to this latter group of participants, here and in the sections to follow. Migrant workers have access to the local healthcare systems in communities in which they work but may be disadvantaged through their participation. Aside from risks to their data, should any unanticipated findings arise, they may experience harms. If their voluntary participation results in brain imaging that reveals incidental findings, such as a medium-sized meningioma or an unruptured aneurysm that only confer a small risk of neurological decline, they may not require immediate clinical treatment but may benefit from longer-term surveillance imaging. Who should bear the cost of these incidentalomas? In Singapore, healthcare financing for citizens and permanent residents is provided via a universal health coverage (UHC) framework (Rajaraman et al., 2020). Subsidized care via this framework is not available to migrant workers and costs must be borne by their employers via mandatory private insurance. However, by this nature, such insurance only provides for basic healthcare costs at levels mandated by governmental policies (Rajaraman et al., 2020). Employers are therefore obliged to cover the costs of acute medical care for their employees. They are not obliged to make provisions for longer term or optional costs, such as surveillance imaging. Conversely, even if migrant workers found to have incidentalomas may themselves opt, following medical counseling, not to have surveillance imaging, knowledge of such seemingly innocuous findings may be disadvantageous to them. Participation in neuroimaging data repositories may therefore provoke unintended economic consequences for migrant workers. If there is a perceived risk to their ability to fully carry out their jobs to the extent they would have been expected to, they may find themselves unable to extend or renew their work passes. Rajaraman et al. (2020) have discussed their vulnerability to termination and repatriation. Work passes are obtained via the sponsorship of individual employers; they are not transferable. Moreover, migrant workers most often fund their own up-front costs (such as agency fees for recruitment, relocation costs and/or travel/interim accommodation). Whilst they would usually recoup such initial outgoings in the medium-term period, an unexpected termination could incur immediate threat of debt. For example, Au (2016), Fillinger et al. (2017), and Rajaraman et al. (2020) have reported that Bangladeshi migrant workers pay between SGD 5,000 and 15,000 in recruitment costs and earn salaries of between SGD 350–800 per month, with SGD 726 per month being the average salary for all migrant workers in Singapore. Thus, a period of employment of more than 1 year is required for migrant workers to begin to fully realize the potential earning power of their contracts.

What are the alternatives? Repositories typically do not have resources to fund follow-up imaging for incidentalomas found on research scans. Lack of provision of surveillance imaging aside, some ethicists have proposed that repositories should necessarily be asked to set aside a portion of their funding as a form of harm mitigation and compensation in the case of unfortunate events (Prainsack and Buyx, 2013). The net result of a migrant worker's participation in a repository may extend to consequences beyond the termination of their livelihood in their host countries. Even after a return to their home countries, implications may follow, such as an inability to obtain personal healthcare insurance to cover the costs of a newfound “known” medical condition.

Which ethical principles should apply here so that the knowledge harvest from a local technological platform would most benefit provision of neuroscience-related healthcare needs in regional LICs and MICs? Do the use of ML/AI methodologies favor the application of one ethical framework over another? Should there be no satisfactory way to address the challenges of including SEA-based migrant workers within a SEA-based neuroimaging data repository, where would they best be represented?

#### 3.1.1 Equity and justice

A key facet of hosting a repository based in SEA would be to provide equitable access in the region to such an innovation and technological advancements that are generated from its use. This would comprise both the (i) increased local capacities from infrastructural improvements, e.g., secure data storage, cloud computing tools and (ii) healthcare innovations based on repository data, e.g., a ML-based neuroimaging triage system for acute brain injury trained on local data. Should the decision for the inclusion or exclusion of migrant workers in local repositories be governed by equity? As per Chadwick and Berg's (2001) definition for genetic databases, equity in repositories refers to “sharing the benefits of research”. By extension, sharing in a neuroimaging-based technical platform requires that equity encompass fair access for LIC and MIC researchers to utilize the repository and also, for migrant workers to participate in the repository for such data to represent the needs of their communities. Yet, as discussed above, migrant workers experience healthcare inequities that participation in the repository may exacerbate. Our example of incidentalomas illustrates that applying equity via the initiative of a repository may cause harms to befall individual participants if the application of equity to wider societal frameworks (e.g., universal healthcare systems) has yet to have been attained. Do we need more than equity?

Here, justice may be a useful additional principle to apply. Justice attends to the dimension of fair distribution of both benefits and burdens of data activities (such as collection, use, and sharing) and also to the issues of equity (Xafis et al., 2019). Its principles of treating individuals and groups fairly, and with respect, clearly favor the inclusion of the migrant worker population. By the application of justice, migrant workers should be included in their host communities as a significant group and be able to participate in society-wide innovations. An example of this view was vaccinations for COVID-19, which were provided at no cost to individuals living in Singapore, regardless of their immigration status. Yet, it can also be argued that, by the use of social justice, migrant worker populations ought to be excluded from local repositories to avoid potential harms arising from their participation. As a population made vulnerable by their immigration status, they may be unduly influenced by fear of reprisals for not participating (as per the above example, in COVID-19) or conversely, enticed by the attractiveness of incentives provided (such as monetary compensation). Equity and justice alone appear to be insufficient as the ethical principles to be applied in the theoretical scenario of a SEA-based repository. In considering both its harms and benefits, what other organizing principle may prove useful?

#### 3.1.2 Solidarity

If neuroimaging data repositories are to meaningfully contribute to the pursuit of Open Science and the benefit of human healthcare, it could be argued that the needs of global communities should necessarily be prioritized over the needs of the individual. In this way, solidarity provides a way of reconciling the ethical responsibilities of repositories to both its sponsors and participants. As per Prainsack and Buyx's (2011, 2013) formulation, solidarity requires that there is willingness on the part of the individual to bear some costs for the benefit of the whole. Whilst societal benefits of neuroimaging data repositories include the potential of ML/AI powered methodologies to transform the delivery of acute neurosurgical care, the standard of care for any one repository participant may not necessarily improve. Neither can there be any expectation of reciprocity. Due to the transitory nature of migrant worker populations, they may not reap the rewards of participation via future healthcare initiatives developed from their datasets.

Does the notion of “solidarity” require that some individuals accept they may bear more costs than others? Rather, it can be argued that the willingness to bear “costs” is the key feature that distinguishes this principle from others that might equally apply to repositories, such as equity and altruism. It is implicit that the costs borne may be disproportionate. In a strike, not all protesters would be at risk of violence or arrest; in a data breach, some might suffer more devastating loss of personal information than others. Would there be a way to better formulate solidarity as the organizing principle for neuroimaging data repositories to include representation from the migrant worker community but without the risk of disproportionate costs to this vulnerable population?

In this regard, it may be helpful to return to Prainsack and Buyx's (2011) definition of solidarity comprising three distinct tiers. The first is distinguished by interpersonal relationships, in which groups of people are willing to bear costs to assist others because they recognize in them a “similarity in a relevant respect”. By this token, it may be plausible to suggest that a SEA-based repository may be more successful at recruiting participants if the sponsors of the said initiative funded mini repositories to be hosted across all partner LIC and MICs instead. To extend the idea of solidarity, it would therefore require that upper MICs and HICs bear disproportionate costs to include participation of LICs and MICs in a SEA-based neuroimaging data repository that would be truly representative of local communities. However, this concept, requiring researchers to concurrently navigate rules, regulations and differing socio-political climates across the region, would likely be both exorbitant in cost and impractical in delivery. A single SEA-based host country, with clear policies regarding the position of its migrant worker population as potential participants, would be far more achievable. Yet, if the relevant ethical principles, such as equity and justice, could be equally employed in the arguments for and against the inclusion/exclusion of migrant workers, what would be deemed a sufficient guiding policy for the governance of local repositories? Is solidarity enough as the dominant principle to determine whether or not migrant workers should be allowed to participate or are the ethical principles in conflict with one another?

#### 3.1.3 Citizenry and universality

Rather than one or two guiding principles, it is possible to resolve such conflicts in the execution of data repositories by the construction of ethical frameworks relevant for the use of big datasets. Indeed, Xafis et al. (2019) have suggested that the use of equity and justice is relevant to this particular application, along with other desirable principles of approach. For the governance framework of the UK Biobank (2007) and Laurie (2017) proposed a more reflexive approach, allowing for the guiding ethical framework of the repository to respond to changes in thinking, e.g., in its handling of incidentalomas, by involving its base of stakeholders, from its sponsors and early participants to members of the public.

Other ethical principles that could apply to a SEA-based repository include citizenry, universality and veracity. Veracity would be most usefully applied to the taking of broad consent to allow for use of data for future purposes such as ML/AI innovations (Lunshof et al., 2008). Citizenry and universality may be used to more strongly argue for the inclusion of migrant workers in this theoretical initiative. In their discussion of genetic databases, Knoppers and Chadwick (2005) suggest two important facets of the utilization of citizenry. The first includes public understanding and consultation about the value of the proposed model toward the advancement of science. This includes promoting the societal viewpoint that migrant workers are a critical group to be represented in the healthcare affairs of the community. Repositories should have a responsibility to ensure the curation of proportionate, ethnicity-based data that is representative of their communities. If the composition and healthcare needs of the local population are reflected, ML/AI-powered healthcare technologies trained on these datasets would be truly transformative. If this is not the case, applications of such technologies in a clinical setting may attract harms, particularly in situations of AI-led triage or decision-making algorithms (Knoppers and Chadwick, 2005). The second, and potentially more critical aspect, is the promotion of a collective identity and engaging the migrant population to partake in efforts toward this from within the communities in which they reside and work in. The notion would be to encourage, within the migrant worker population, the viewpoint that they belong to the community group and should participate in local efforts of their host countries. In this regard, it would be crucial to include migrant workers that represent communities in the region, especially LICs and MICs in a SEA-based neuroimaging data repository. The risk that, should they not be represented in regional initiatives, they would not be included anywhere in global projects would surely outweigh the benefit to individuals of avoiding potential harms.

Knoppers and Chadwick (2005) also expand upon the concept of universality that includes characterization of human data as a shared resource, from which a wide range of applications toward benefit-sharing would be possible. Participants in the repository will receive no part of the profits from technologies created based on this shared knowledge. Universality promotes the viewpoint that efforts to amass representative local datasets are important toward influencing the production of global goods of greater value to the world community at large.

However, even the transparency and thoughtfulness of an ethical framework comprising the principles above may not be sufficient to mitigate harms to migrant workers arising from a SEA-based neuroimaging data repository. Instead of excluding them entirely, one approach to address such shortcomings would be the establishment of funds to compensate migrant workers for any harms (e.g., potential fallout form incidentalomas) that occur via their participation in repositories. Another approach would be to consider promoting ethical usage and development of technological tools leveraging on the data amassed via neuroimaging data repositories. This is consistent with strategies toward “Responsible AI”, which we discuss further below.

## 4 The mini AI ethics experiment

The need for the concept of “Responsible AI” has arisen due to the rapid growth of ML/AI technologies and their transformative translational potential. In healthcare applications, misapplied utility of data and the development of biased algorithms is an emerging area of concern. The issue of disproportionate datasets (such as over- or under-representation of cohorts, inadequate sample variables or data points, or biased selection of disease or outcome markers), lies at one end of the spectrum. At the other is the risk of exploitation of sensitive data and the unethical use of brain metrics derived from the repository to promote scientific racism. Using standard statistical tools, variations in brain structures have been previously used to imply significant racial differences in intelligence (Evans, 2018). ML/AI-based methodologies may incorporate such rhetoric found in published literature (e.g., the use of large language models such as ChatGPT to analyze imaging reporting data) or may accelerate bias, racism, or sexism (Vallance et al., 2023) if contextual data for variations in brain structures are not collected. Environmental threats may trigger brain responses, resulting in changes in structural metrics over time. The brain and its functions may be remodeled by the effects of endemic stressors, such as infectious diseases (Eppig et al., 2010), but these effects may appear correlated to characteristics of ethnicity or race, and these factors falsely linked to causality or inherent genetic differences. Similarly, the burden of chronic illnesses may be shaped by healthcare inequities, such as access to subspecialist care or lack thereof, producing differing outcomes, both within the local community and across global populations. Such variations require proper training, using strategies to mitigate bias, to avoid inappropriate interpretations of differences inherent across ethnicity-specific datasets that may be impacted by socio- or geo-political determinants of health and disease.

“Responsible AI” has emerged as a movement in response to such critique. The concept has been shaped both by will from within the ML/AI developers' community and without it, driven by political pressure to regulate potential harms from tech innovations (McCabe, 2023). For example, Microsoft, which has heavily invested in the generative AI technologies behind ChatGPT (Metz and Weise, 2023), has stated its commitment to the advancement of AI driven by ethical principles. Yet, the power to develop “Responsible AI” tools has largely been left in the purview of commercial tech giants and interested developers. There is a hidden aspect to ethical AI practices to which others hold the key. The curation of high quality, representative datasets upon which lie the foundation of ML/AI algorithms is possibly of far greater importance in influencing the outcome of such work. There is little understanding in the scientific and medical communities about best practices in data curation to prepare for ML/AI methodologies. What if the gap between AI and clinical researchers could be tested to understand concerns of mis-utility of data? Would it be possible to use ML/AI methodologies to interrogate its own gaps and shortcomings in the interpretation of clinically disproportionate or under-representative datasets? Here, we present a mini-experiment to illustrate a strategy we term “AI Ethics”.

As a case example of an under-representative dataset, we used a pilot sample from an ongoing study of long COVID. Our hypothesis for the study was that the initial presentation of COVID-19 with or without anosmia conferred differing implications for the longer-term development of neurological risks. In analyzing the pilot study, we found some promising results. However, there are two current ways in which our under-representative dataset may contribute to scientific misinterpretation:

(i) there is a vast variation in the incidence of anosmia across the global populations—are our results generalizable? and
(ii) our sample is small and subject to the usual considerations in the analysis of modest datasets. How can we be sure our findings are robust?

### 4.1 Methodology of mini experiment

We have previously published on global variations in the presentation of anosmia, ageusia and neurological risks in COVID-19 (Kumar et al., 2021). In our previous work and in the earlier methods section above, we have described our methodologies for the derivation of brain metrics, in particular, the use of 3D structural segmentation for brain volumes and diffusion tensor imaging (DTI), for the examination of brain injury signatures (Lock et al., 2019; Soon et al., 2021). For the purposes of this mini experiment in AI ethics, we used ML/AI to model and extrapolate the pilot dataset to look for potential areas where findings converged or diverged based on the proportionality of cohorts represented.

**Figure 3:**
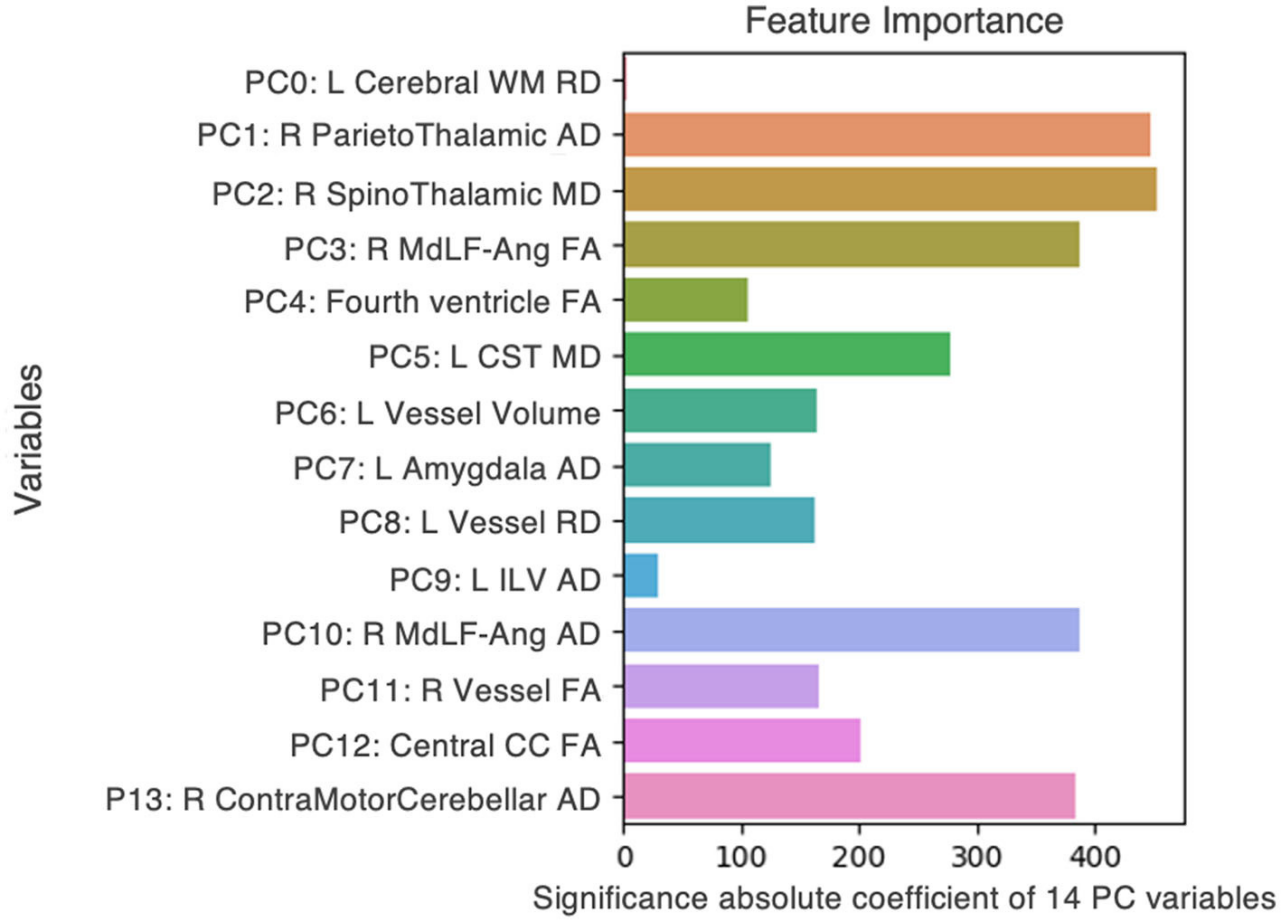
Fourteen features, from DTI metrics and structural volumes, that best distinguished between non-anosmic and anosmic COVID recoverers in the pilot dataset. L, left; R, right; FA, fractional anisotropy; MD, mean diffusivity; AD, axial diffusivity; RD, radial diffusivity; CC, corpus callosum; Cerebral WM, cerebral white matter; CST, corticospinal tract; ILV, inferior lateral ventricle; MdLF-Ang, middle longitudinal fasciculus–superior angular gyrus component.

Firstly, we interrogated the actual pilot dataset [*n* = 17; eight non-anosmic (47%), nine anosmic (53%)], using a clustering model of ML generated in-house to discover the principal components that best distinguished non-anosmic vs. anosmic COVID recoverers. After classification, the fourteen best features were chosen (Figure 3). Next, we used bootstrapping to model three hypothetical datasets. We randomly resampled from the original pilot dataset to created multiple new samples that amplified the dataset to produce a sample three to four times the original, whilst attempting to represent differing proportions of non-anosmic vs. anosmic cohorts found across global populations. The final hypothetical datasets (3.8 to 4.0-fold amplified from the original) were:

(1) The matched dataset (*n* = 68; 32 non-anosmic, 36 anosmic) 47/53% proportions of non-anosmic/anosmic as per pilot.
  This dataset was somewhat artificially balanced, as per standard scientific practice, to simulate our pilot COVID-19 dataset; this allowed us to compare our pilot COVID-19 dataset, to compare equivalent numbers of different groups but is not necessarily reflective of actual incidence of groups in the local population.
(2) The predominant non-anosmic dataset (*n* = 66; 48 non-anosmic, 18 anosmic).
  This dataset is 73% disproportionate in favor of non-anosmic cohorts but may be representative of the SEA populations, e.g., the lower incidence of anosmia in Singapore.
(3) The predominant anosmic dataset (*n* = 66; 16 non-anosmic, 50 anosmic).
  This dataset is 76% disproportionate in favor of anosmic cohorts but may be more representative of worldwide populations (Lechien et al., 2020; von Bartheld et al., 2020; Kumar et al., 2021).

**Figure 4:**
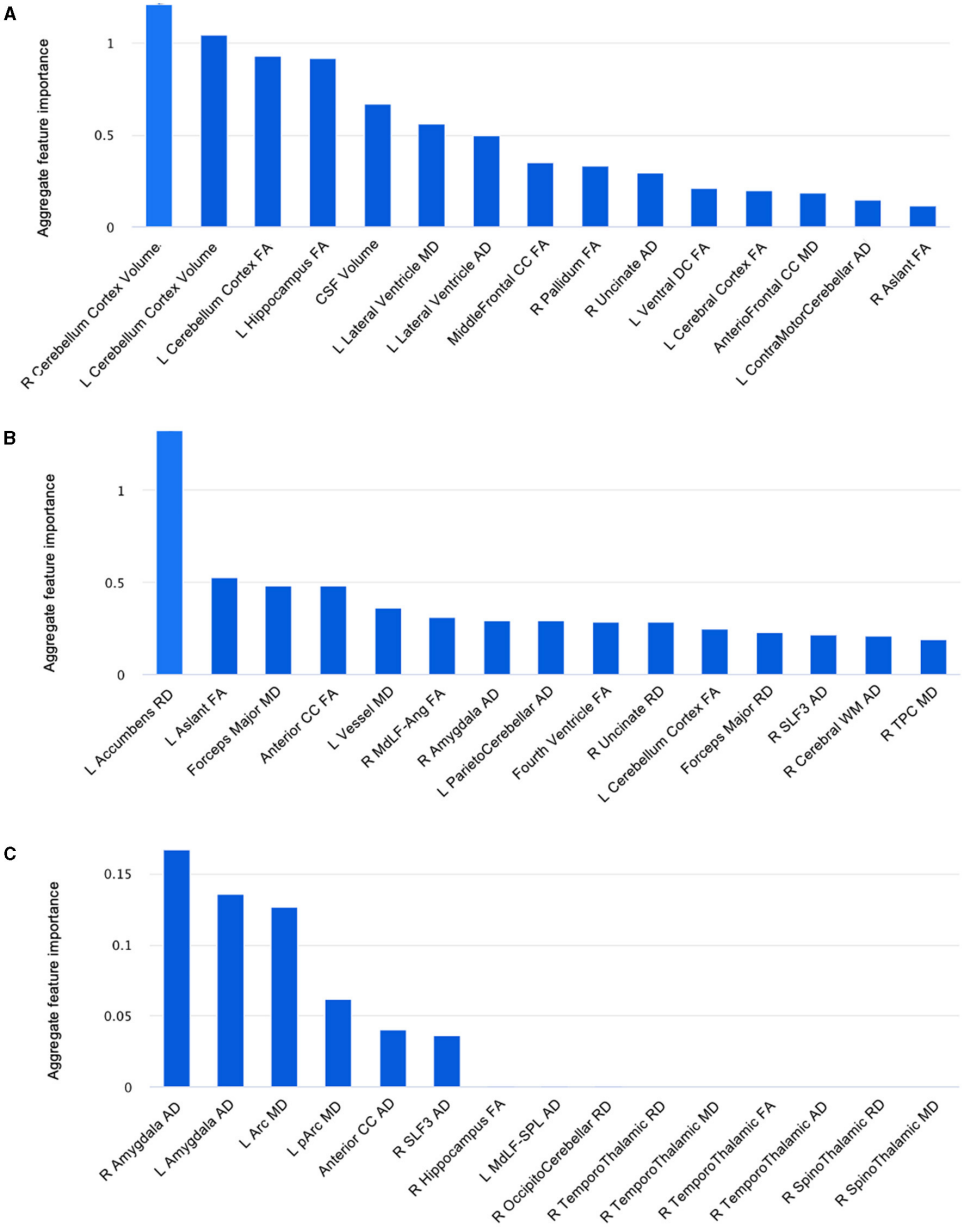
Fifteen features, from DTI metrics and structural volumes, that best distinguished between non-anosmic and anosmic cohorts in the bootstrapped **(A)** matched dataset, **(B)** predominant non-anosmic dataset, and **(C)** predominant anosmic dataset. L, left; R, right; FA, fractional anisotropy; MD, mean diffusivity; AD, axial diffusivity; RD, radial diffusivity; Arc, arcuate fasciculus; CC, corpus callosum; CSF, cerebrospinal fluid; MdLF-Ang, middle longitudinal fasciculus–superior angular gyrus component; MdLF-SPL, middle longitudinal fasciculus-superior parietal lobule component; pArc, posterior arcuate fasciculus; SLF, superior longitudinal fasciculus; TPC, temporo-ponto-cerebellar; Ventral DC, ventral diencephalon.

We then performed ML using Microsoft's Azure Machine Learning Studio, a commercially available cloud computing tool with the capacity to build, train and deploy models for a variety of applications.^3^ Following training using the original pilot dataset, we tested the three hypothetical COVID-19 datasets to generate the best classification models for each. We then extracted the top fifteen features by their importance and compared these amongst both the hypothetical and actual pilot samples (Figure 4).

## 5 Results

In summary, we found that the proportions of the cohorts represented in each dataset altered not only the relative importance of top key features distinguishing between them but the presence or absence of even predominant key features. For example, 3D-segmented structural volumes were only relevant as key features in the pilot and matched hypothetical dataset, that represented a similar cohort that had been derived by randomly resampling the pilot dataset in the closest proportions to the original experiment by four-fold. The key volumes of interest were left vessel volume (pilot) (Figure 3) and right and left cerebellar cortex volumes (matched dataset) (Figure 4A). For the remainder of the features for the pilot and matched datasets, as well as all the key features for both of the other hypothetical datasets, DTI measures were best at correctly classifying the non-anosmic vs. anomic cohorts. DTI metrics of the amygdala (right and/or left axial diffusivity) were a key feature in classifying cohorts in the pilot (Figure 3) and both disproportionate datasets (predominant non-anosmic or anosmic; Figures 4B, C, respectively). This feature was strongest for the predominant anosmic cohort. In addition, DTI metrics for structures related to CSF and the ventricles (including the corpus callosum white matter tract) were key features in common between the pilot and all three hypothetical datasets (Figures 3, 4A–C).

## 6 Discussion

**Figure 5:**
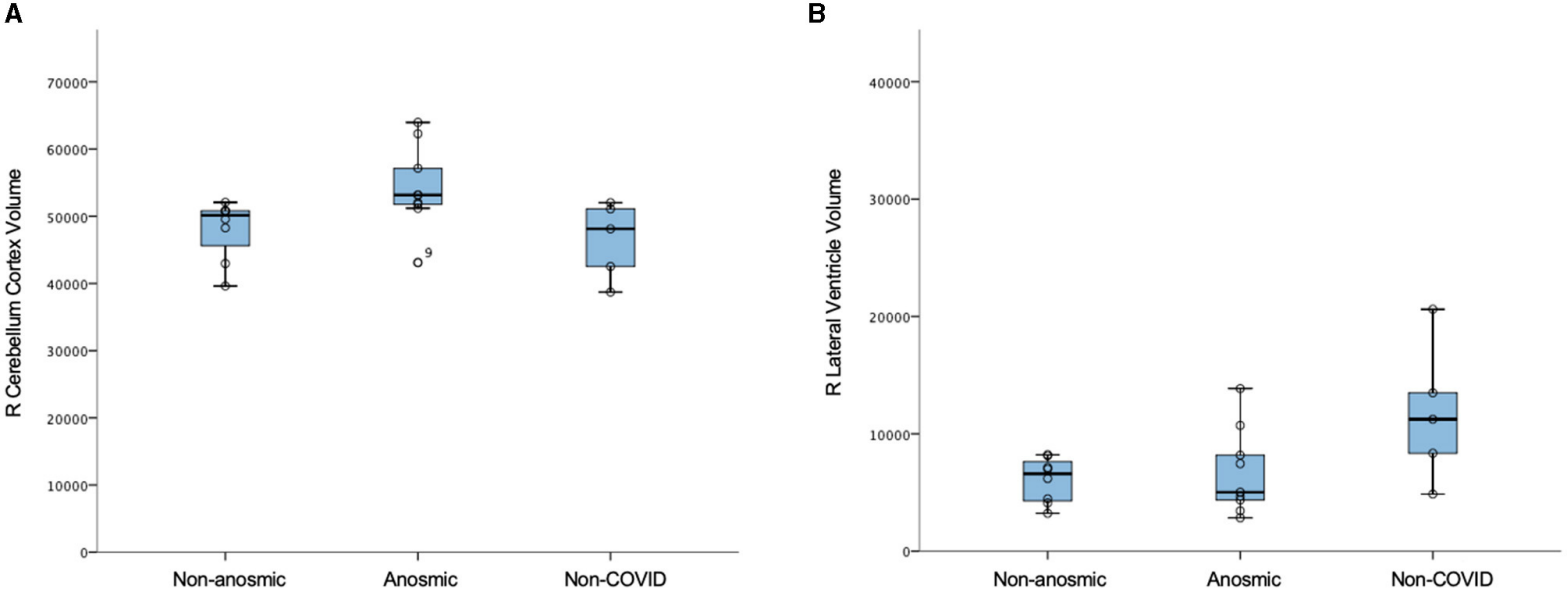
Structural volumes (voxels) in non-anosmic and anosmic COVID-19 recoverers, compared to older non-COVID cohorts; for the **(A)** right cerebellum cortex and **(B)** right lateral ventricle.

**Figure 6:**
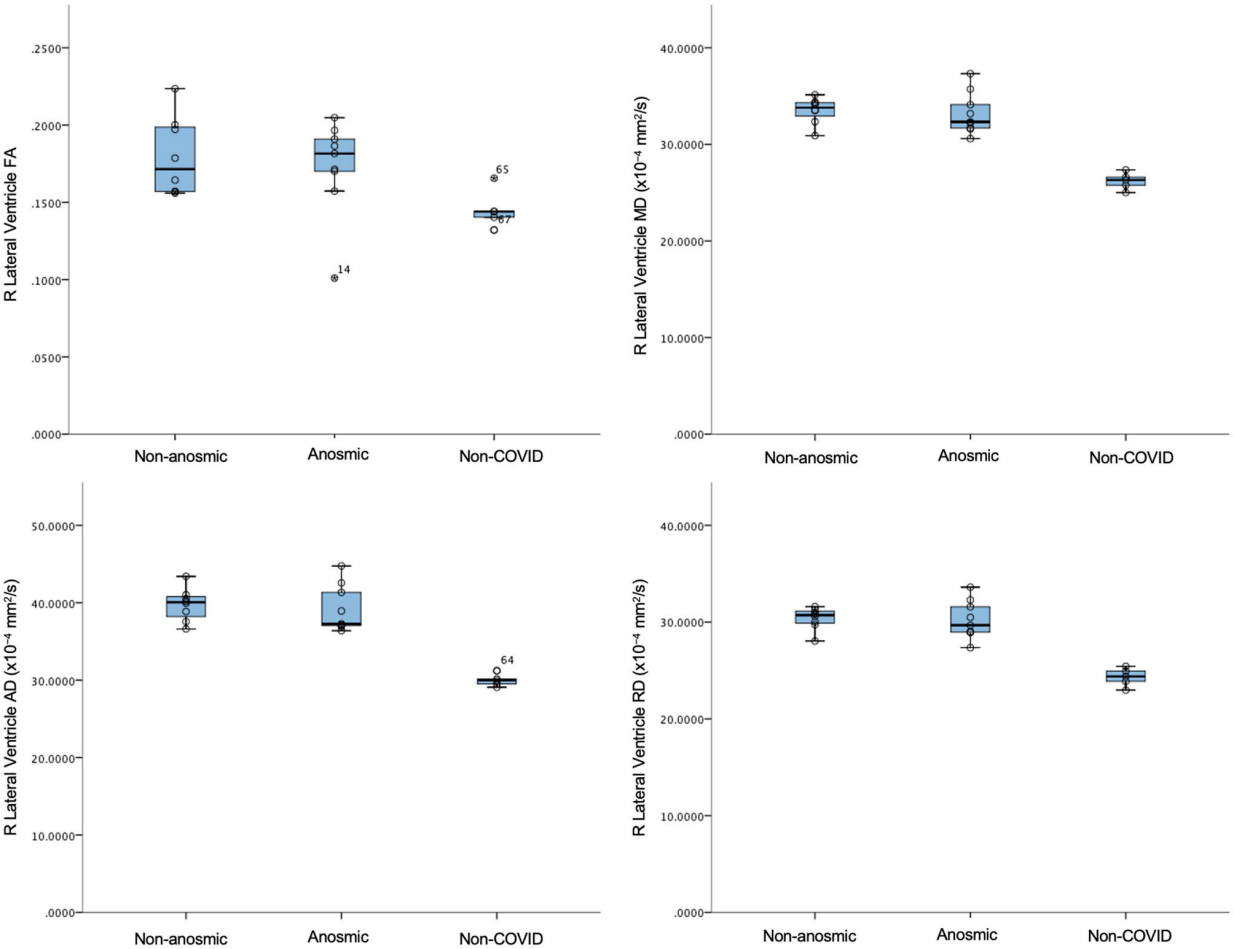
DTI metrics for the right lateral ventricle in non-anosmic and anosmic COVID-19 recoverers, compared to older non-COVID subjects.

In this mini AI ethics experiment, we sought to use ML/AI tools to predict their behavior when presented with both balanced and disproportionate datasets. In so doing, we were able to model the hypothetical effects of proportional representation of differing patient cohorts on brain structural and microstructural findings in a projected dataset of larger numbers. We found that structural volumes were only helpful in classifying cohorts that were well-balanced and matched. In general, volumes for the cerebellar cortex were lower for the predominantly non-anosmic cohort, approaching the range of a non-COVID cohort of older individuals (Figure 5). The most consistent group of key features that distinguished the predominantly non-anosmic from the predominantly anosmic cohort comprised structures related to CSF and the ventricles. DTI metrics for the latter (in the matched dataset; tissue signatures consistent with that expected of periventricular white matter) and the corpus callosum demonstrated higher fractional anisotropy (FA), driven by higher axial diffusivity (AD), in COVID-19 recoverers vs. older non-COVID subjects (Figure 6). In our previous work, this DTI profile was suggestive of white matter distortion by stretch/compression (Keong et al., 2022). Confirmation of this pattern here would require the examination of a larger, actual dataset, rather than via hypothetical modeling. However, unlike our work in the condition of Normal Pressure Hydrocephalus (NPH), which is characterized by ventriculomegaly, the white matter injury in COVID-19 recoverers is not known to be associated with an abnormal increase in ventricular volumes. Indeed, in the predominantly non-anosmic cohort, where this DTI profile was strongest, we found that lateral ventricular volumes were the smallest compared to the predominantly anosmic cohort and older non-COVID subjects (Figure 5). This suggests that another mechanism occurring in the interstitial spaces surrounding the periventricular/adjacent white matter may be contributing to tissue distortion in this specific region.

Another key feature of interest was the amygdala, where we found alterations in DTI metrics, particularly in axial diffusivity (AD). In general, there were higher axial diffusivities in both COVID-19 cohorts compared to older controls. Here, age may be a factor, but the consistency of the representation of the amygdala as a key feature across both disproportionate datasets and the pilot sample suggests a more meaningful link to COVID-19. The effect is lost in the well-balanced, matched dataset. However, in this cohort, other gray matter structures, such as the hippocampus and pallidum, still feature. The top feature in the predominantly non-anosmic dataset was the left accumbens radial diffusivity. In general, the COVID-19 cohorts had increased axial, mean and radial diffusivities (AD, MD, and RD, respectively) and a trend toward lower structural volumes compared to older controls. These findings cannot be easily attributable to age. Conversely, in the predominantly anosmic cohort, the effect of the axial diffusivity metric of the amygdala was so strong that the structure was represented bilaterally as the top two key features in the classification of this cohort. Taken together, the findings above suggest two areas of exploratory interpretation:'

(i) That the amygdala axial diffusivity metric may be relevant as a comparator for global COVID-19 cohorts, due to its relevance to both extremes of the spectrum of rates of non-anosmia vs. anosmia known in the presentation of the disease.
(ii) In the context of COVID-19, the biological response to the allostatic load of stress could plausibly involve remodeling of the amygdala. Pathophysiological changes in the amygdala have been shown in other chronic diseases (Hu et al., 2022). The mechanism for these changes is thought to be neuro-inflammation; inflammation is also postulated to be the trigger for long COVID across multiple organ systems. This suggests that the hypothetical datasets may provide directions that may be useful to examine further via an actual, larger dataset of recoverers.

### 6.1 Limitations

The mini experiment explored a pilot sample and three hypothetical samples, amplified by three to four-fold the original dataset. This approach is clearly inferior to amassing a real dataset of the same magnitude and interrogating its trends. As the pilot sample is small and the other datasets hypothetical, we did not perform statistical comparisons but rather, examined the data qualitatively. The controls provided were older and from a pre-COVID cohort. None of these methods are ideal and we intend to correct such shortcomings with future work on larger datasets. Nevertheless, the main thrust of the mini experiment was to understand if ML/AI methodologies could be utilized toward modeling disproportionate datasets to understand risk of bias, generalizability and applicability of such models. More work is needed toward honing this new strategy.

## 7 Conclusion

In this paper, we examined the concept of neuroimaging data repositories as a resource for a global tech platform, whose innovations are driven by ML/AI methodologies. Firstly, we approached a theoretical experiment of the creation of a SEA-based repository to consider ethical concerns arising from such an innovation. Secondly, we utilized ML/AI methodologies to develop an approach to AI ethics for the purposes of examining how the projected findings are influenced by the levels of proportionate representation in disease datasets. Further work is required to expand the questions and directions raised here into a reproducible strategy toward AI ethics.

## Data availability statement

The original contributions presented in the study are included in the article/Supplementary material, further inquiries can be directed to the corresponding author.

## Ethics statement

The studies involving humans were approved by SingHealth Centralised Institutional Review Board (CIRB). The studies were conducted in accordance with the local legislation and institutional requirements. The participants provided their written informed consent to participate in this study.

## Author contributions

CL: Formal analysis, Investigation, Project administration, Writing – review & editing, Writing – original draft. NT: Formal analysis, Writing – review & editing. IL: Writing – review & editing, Investigation. NK: Conceptualization, Formal analysis, Investigation, Methodology, Visualization, Writing – original draft, Writing – review & editing.

## Footnotes

^1^*Health Insurance Portability and Accountability Act 1996* (United States) Pub. L. No. 104-191. Available online at: https://www.govinfo.gov/app/details/PLAW-104publ191/.

^2^*Data Protection Act 2018, c. 12* (United Kingdom) s 3(10). Available online at: https://www.legislation.gov.uk/ukpga/2018/12/contents.

^3^Available online at: https://azure.microsoft.com/en-gb/products/machine-learning/#overview (last accessed November 1, 2023).
