# Supplementary material for: Neuroimaging data repositories and AI-driven healthcare—Global aspirations vs. ethical considerations in machine learning models of neurological disease
Source: Front Artif Intell. 2024 Feb 19;6:1286266. doi: 10.3389/frai.2023.1286266 (PMC10910099; doi:10.3389/frai.2023.1286266)
Supplement: Supplementary file 1 [file Table_1.DOCX]

Supplementary Material

Neuroimaging data repositories and AI-driven healthcare – global aspirations vs. ethical considerations in Machine Learning models of neurological disease

# Supplementary Table

**Supplementary Table 1**. Applications on brain-life.io used for diffusion MRI processing

| Application | Link to online app |
| --- | --- |
| Anatomical alignment | <https://doi.org/10.25663/brainlife.app.273> |
| Brainlife wrapper for Freesurfer | <https://doi.org/10.25663/brainlife.app.462> |
| Freesurfer statistics | <https://doi.org/10.25663/brainlife.app.272> |
| dMRI preprocessing | <https://doi.org/10.25663/bl.app.68> |
| MRTrix3 | <https://doi.org/10.25663/brainlife.app.319> |
| Cortex tissue mapping | <https://doi.org/10.25663/brainlife.app.381> |
| Compute subcortical diffusion measures | <https://doi.org/10.25663/brainlife.app.389> |
| Compute cortical diffusion measures | <https://doi.org/10.25663/brainlife.app.483> |
